# Total Parenteral Nutrition Successfully Treating Black Esophagus Secondary to Hypovolemic Shock

**DOI:** 10.1155/2017/4396870

**Published:** 2017-06-18

**Authors:** Tony S. Brar, Richard Helton, Zareen Zaidi

**Affiliations:** Department of Internal Medicine, University of Florida, Gainesville, FL, USA

## Abstract

We present a patient who developed black esophagus secondary to hypovolemic shock and was placed on total parenteral nutrition for three weeks after hospital discharge. The area of interest is the multimodal approach used in treatment of this noncompliant patient. Even with a high mortality rate, this case illustrates a successful outcome of a patient who responded to appropriate immediate therapy resulting in complete resolution of the necrosis with no further development of complications.

## 1. Introduction

Acute esophageal necrosis (AEN), also known as necrotizing esophagitis or black esophagus, is a rare syndrome affecting the distal esophagus, terminating near the gastroesophageal junction (GEJ) [1, 2]. Although biopsy is generally recommended, it is not required to make the diagnosis. In an autopsy series, black esophagus had an estimated prevalence of 0.2 percent, while in an endoscopy series the prevalence was as low as 0.001 percent [3, 4]. The exact etiology is not very clear, but a hypothesis exists stating that an initial event decreasing vascular flow results in ischemia predisposing the esophageal mucosa to the reflux of pepsin and acid resulting in topical injury ultimately leading to necrosis [5]. Even with supportive care and treatment of the underlying illness, mortality rates are still near 35 percent [3]. We present a very interesting case of a patient in hypovolemic shock secondary to a gastrointestinal (GI) bleed with black esophagus that, due to contraindications, required total parenteral nutrition (TPN) resulting in complete resolution of the esophageal mucosa necrosis.

## 2. Case Presentation

A 59-year-old female with a past medical history of alcohol abuse and depression was brought into the hospital by emergency medical services (EMS) after being found down at her home. The patient was significantly altered and unable to provide an accurate history; therefore history was obtained through EMS and her spouse. She was found covered in her own emesis, feces, and urine. She was in hypovolemic shock being tachycardic, hypotensive, and unable to follow commands. She was likely hypoventilating due to alcohol intoxication and had an acute on chronic GI bleed resulting in significant volume loss. On initial presentation she was acidotic with a pH of 7, pCO2 of 32 mmHg, pO2 of 33 mmHg, and bicarbonate of 8 mmol/L. She was immediately intubated for airway protection and placed on broad-spectrum antibiotics. Due to inadequate response to fluid resuscitation she required cardiac support with vasopressors.

**Figure 1:**
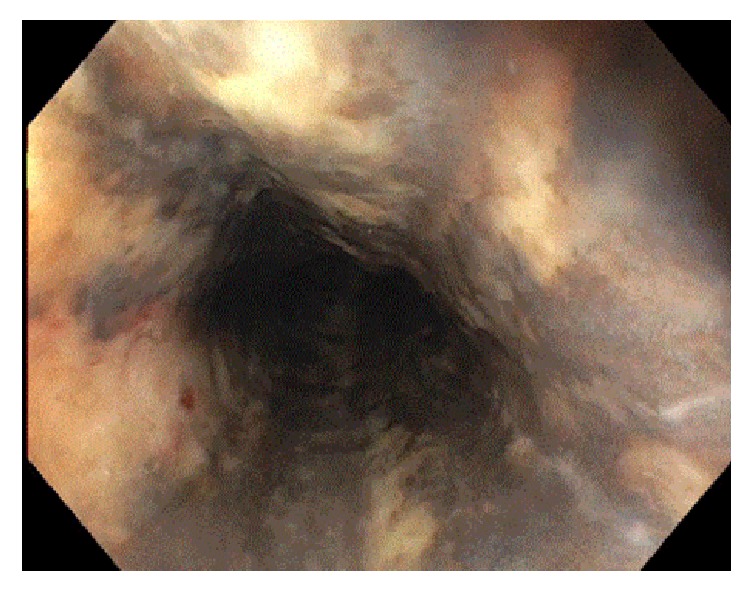
Initial EGD upon presentation showing severe necrosis in the middle to lower third of the esophagus.

She had a mild transaminitis with AST 50 U/L and ALT 22 U/L. Her alkaline phosphatase was 145 U/L with total bilirubin of 2.3 mg/dL and direct bilirubin of 1.2 mg/dL. A CT scan of her chest, abdomen, and head revealed hepatic steatosis, but no acute process in chest, abdomen, or pelvis and no acute intracranial abnormalities. Her urine drug screen was negative and ethyl level was positive. She had initial hemoglobin of 7.3 g/dL and hematocrit of 24.8% and was guaic positive. She was started on pantoprazole and octreotide infusions. An upper endoscopy (EGD) was performed showing severe mucosal changes characterized by necrosis at the middle and lower thirds of the esophagus (Figure 1). This was consistent with black esophagus. There was diffuse moderately erythematous mucosa in the entire stomach. Only one nonbleeding gastric ulcer was found in the gastric antrum. She was admitted to the intensive care unit (ICU), placed on the clinical institute withdrawal assessment for alcohol (CIWA) protocol, and closely monitored.

After a couple days her mentation improved and she was able to be extubated and weaned off her vasopressors. She was taken off her broad-spectrum antibiotics as her cultures showed no growth and she was hemodynamically stable. However she continued to remain at high aspiration risk and had difficulty swallowing minimal liquids. Given the extent of her esophageal necrosis, a nasogastric tube (NGT) was contraindicated. Interventional radiology was consulted and stated they would not be able to place a gastric tube (G-tube) for nutrition since a NGT was required for proper placement. General surgery was consulted and deferred placing a G-tube as they felt patient had a high risk of perforation. As the patient continued to show improvement nutrition became of the utmost importance.

**Figure 2:**
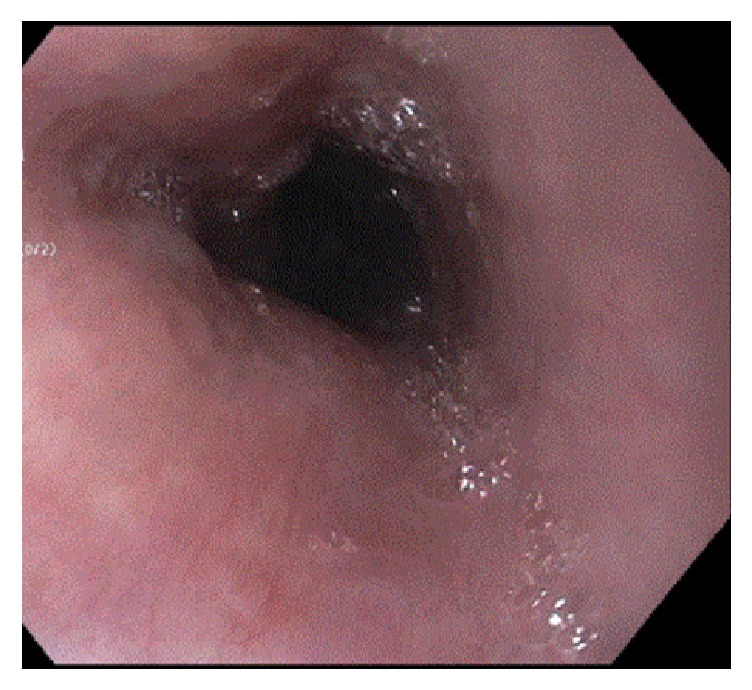
Follow-up EGD 3 weeks after hospital discharge showing normal mucosa in the middle to lower third of esophagus.

We then placed a peripherally inserted central catheter (PICC) and initiated TPN. She refused to go to rehab and was eventually able to be discharged home with home care for her TPN and scheduled for follow-up EGD in 3-4 weeks. Her repeat EGD showed normal esophagus and duodenum (Figure 2). She was transitioned to a soft diet for one week and tapered off her TPN. She remained on oral pantoprazole and ultimately completely recovered from her episode of the black esophagus.

## 3. Discussion

This represents an interesting case in literature of a patient who developed black esophagus secondary to hypovolemic shock and was placed on TPN for a total of three weeks after hospital discharge. Fortunately this resulted in complete resolution of the necrosis. It is known that timely patient stabilization and fluid resuscitation play pivotal roles in properly managing these difficult patients [6]. Furthermore, as seen from other published cases on the topic, acid suppression with high dose intravenous proton pump inhibitors, nil-per-os (NPO) restriction, and glycemic control should immediately be initiated [7]. However, with regard to adequate nutrition for patients, the management tends to be up to the discretion of the clinical provider.

Fortunately our patient did not have the immediate local complications commonly associated with the syndrome which include perforation and mediastinitis [8]. However, due to the high risk of decompensation in our patient, G-tube placement was deferred. With the proper addition of dextrose, multivitamins, trace elements, amino acids, and electrolytes, the patient received sufficient TPN that helped provide time for mucosal healing. This case illustrated the multimodal approach that is necessary to treat a complicated condition. She had a history of noncompliance and the decision to pursue home TPN therapy was difficult; however, it did prove to be successful. Each patient is different, but it is important to take into account the entire situation and sometimes hope for the best as not all cases result in positive outcomes.
